# Pathogenic effects of Leu200Pro and Arg387His VRK1 protein variants on phosphorylation targets and H4K16 acetylation in distal hereditary motor neuropathy

**DOI:** 10.1007/s00109-024-02442-8

**Published:** 2024-03-30

**Authors:** Aurora Campos-Díaz, Patricia Morejón-García, Eva Monte-Serrano, David Ros-Pardo, Iñigo Marcos-Alcalde, Paulino Gómez-Puertas, Pedro A. Lazo

**Affiliations:** 1 Molecular Mechanisms of Cancer Program, Instituto de Biología Molecular y Celular del Cáncer, Consejo Superior de Investigaciones Científicas (CSIC), Universidad de Salamanca, 37007 Salamanca, Spain; 2 Instituto de Investigación Biomédica de Salamanca (IBSAL), Hospital Universitario de Salamanca, 37007 Salamanca, Spain; 3 Molecular Modeling Group, Centro de Biología Molecular Severo Ochoa, CBMSO (CSIC-UAM), 28040 Madrid, Spain

**Keywords:** Chromatin, DNA damage, Epigenetics, Histone acetylation, Motor neuron diseases, VRK1

## Abstract

**Abstract:**

Rare recessive variants in the human *VRK1* gene are associated with several motor neuron diseases (MND), such as amyotrophic lateral sclerosis, spinal muscular atrophy, or distal hereditary motor neuropathies (dHMN). A case with dHMN carrying two novel VRK1 gene variants, expressing Leu200Pro (L200P) and Arg387His (R387H) variant proteins, identified that these protein variants are functionally different. The Leu200Pro variant shares with several variants in the catalytic domain the loss of the kinase activity on different substrates, such as histones, p53, or coilin. However, the distal Arg387His variant and the distal Trp375* (W375X) chinese variant, both located at the end of the low complexity C-terminal region and proximal to the termination codon, retain their catalytic activity on some substrates, and mechanistically their functional impairment is different. The L200P variant, as well as most VRK1 pathogenic variants, impairs the phosphorylation of BAF and histone H4K16 acetylation, which are required for DNA attachment to the nuclear envelope and chromatin accessibility to DNA repair mechanisms, respectively. The R387H variant impairs phosphorylation of H2AX, an early step in different types of DNA damage responses. The functional variability of VRK1 protein variants and their different combinations are a likely contributor to the clinical phenotypic heterogeneity of motor neuron and neurological diseases associated with rare VRK1 pathogenic variants.

**Key messages:**

- VRK1 variants implicated in motor neuron diseases are functionally different.
- The L200P variant is kinase inactive, and the R387H variant is partially active.
- VRK1 variants alter H4K16 acetylation and loss of coilin and BAF phosphorylation.
- VRK1 variants alter Cajal bodies and DNA damage responses.
- VRK1 variant combination determines the neurological phenotype heterogeneity.

**Supplementary Information:**

The online version contains supplementary material available at 10.1007/s00109-024-02442-8.

## Introduction

Functional alterations of motor neurons are associated with several neurological diseases. Genetic heterogeneity is present and several genes have been implicated in these diseases [1, 2]. Rare variants of the human *VRK1* gene are associated with a heterogeneous group of neurological diseases, affecting motor neurons and the peripheral nerves, sensory and sensory-motor. Among these diseases are spinal muscular atrophy (SMA), amyotrophic lateral sclerosis (ALS), distal hereditary motor neuropathies (dHMN), and Charcot-Marie-Tooth (CMT) disease [3–9]. All *VRK1* gene variants associated with these diseases are recessive and their clinical phenotypes are manifested either in homozygous or compound heterozygous individuals [3–9]. The evolution of these diseases affecting motor neurons is slowly progressive, often starting early in life, and their clinical manifestations are initially distal. The slowly progressive phenotype in non-dividing cells, such as neurons, indicates that the pathogenic mechanism is a likely consequence of a slow accumulation of axonal alterations in neurons with time, and that may also be associated with the length of the axons, which requires a larger transport along long axons. Furthermore, alterations in histone epigenetic modifications have also been implicated in some motor neuron diseases (MND), such as SMA [10, 11] and ALS [12, 13].

VRK1 is mostly a chromatin/nuclear kinase implicated in the regulation of several processes such as chromatin remodeling [14, 15], cell cycle progression [16, 17], DNA damage responses [18–22], and assembly of Cajal bodies [23]. Alterations in any of these three functions, chromatin remodeling, Cajal bodies, and DNA damage responses are very relevant pathogenic mechanisms in non-dividing neurons. Chromosomes are attached to the nuclear envelope by its binding to BAF, which is regulated by its phosphorylation by VRK1 that permits its detachment from the nuclear envelope [24–26]. Furthermore, chromatin remodeling is associated with histone acetylation, such as H4K16 acetylation (H4K16ac), a common mark to many processes, such as transcription, replication, or DNA damage responses. The acetylation of H4K16 is mediated by several lysine acetyl transferases (KAT) such as Tip60 [27–30] or MOF [31]. VRK1 directly interacts and phosphorylates Tip60 promoting its translocation to chromatin and its transacetylase activity in the DNA damage response [27, 30]. VRK1 depletion also alters several nuclear phosphoproteome pathways in the response to DNA damage [32].

VRK1 pathogenic variants are located within three clusters in the protein sequence [33]. Two clusters, I and II, are located in the kinase N-terminal domain immediately after the ATP binding site or the catalytic site respectively. The third cluster (III) is located near the end of the low-complexity C-terminal regulatory region [33]. The VRK1 C-terminal region is very flexible and can have multiple alternative conformations [34]. This C-terminal region folds and interacts with the N-terminus, inhibiting the kinase activity, and with other proteins determining the complexes in which it participates within the nucleus [15, 33]. Unless the patients are siblings, there is not a single case with the same combination of VRK1 variants [33].

In this report, we have characterized two new VRK1 variants ((c.599 T > C; p.Leu200Pro) [35] and (c.1160G > A; p.Arg387His) [35, 36]), in the context of their pathogenic mechanism that take place in non-proliferating cells. As well as the common effect of several additional pathogenic VRK1 protein variants, representing the three clusters in the VRK1 amino acid sequence. Cluster I is located next to the ATP binding site, cluster 2 next to the catalytic site, both within the kinase domain, and cluster 3 near the end terminus of the protein sequence [33]. We have focused the study on effects regarding the phosphorylation of specific substrates, as well as on histone acetylation and DNA damage response and other nuclear regulatory functions that can have relevant consequences in the neuro-pathogenic mechanism. The two new variants identified in the patient have different roles, one affects the kinase activity, while the other affects regulatory processes such as histone acetylation or alteration of protein complexes in which the VRK1 protein participates. The differences between the two new VRK1 variants, or other variants, which in patients have different variant combinations, can be the determining factor for the heterogeneity of the specific neurological phenotype manifested by individual patients.

## Results

### Structural prediction of VRK1 L200P and R387H pathogenic protein variants

**Fig. 1:**
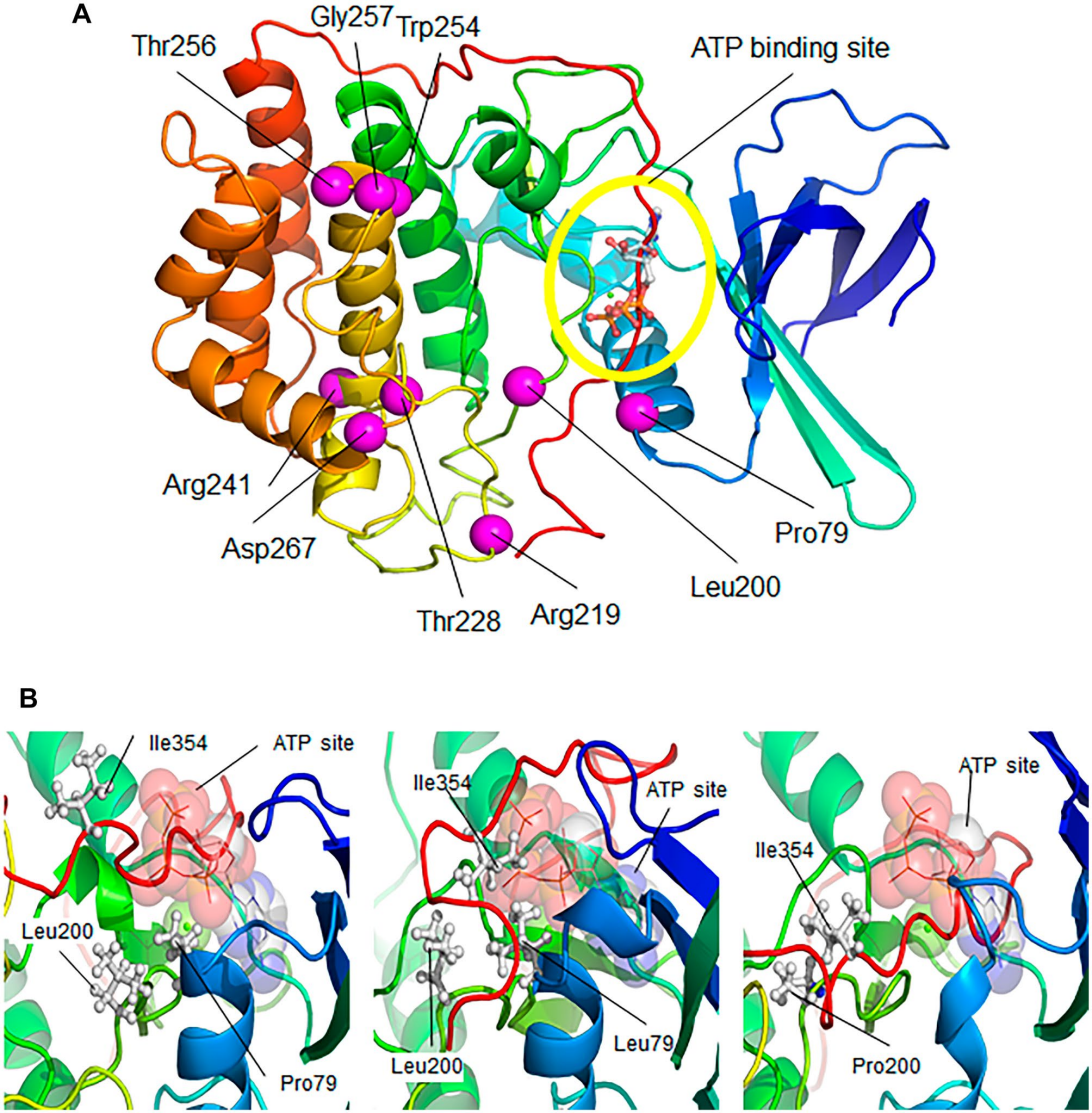
Structural modeling of the human VRK1-L200P variant protein. **A** Structure of the wild-type VRK1 activation loop after 200 ns of free molecular dynamics. **B** In the simulation of the wild-type (wt) enzyme, the hydrophobic amino acids Pro79 and Leu200 (**B**, left) interact closely, being located away from the Ile354 residue, which allows some flexibility of the C-terminal tail of the enzyme, facilitating the regulation of auto-inhibition through the cycle of occupancy and release of the active center (ATP site in **B**). In the case of the Pro79Leu and Leu200Pro variants, the result of molecular dynamics simulation of both mutations is a closer contact of Ile354 with Leu79 and Leu200 (Pro79Leu variant, **B**, center) or of Ile354 with Pro200 (Leu200Pro variant, **B**, right). In both cases, this closer interaction would negatively impact the opening of the active center and thus VRK1 activity

Initially, we performed a molecular modeling of the new pathogenic protein variants to identify their impact on VRK1 structure, and their functional consequences. The structural localization of the variants studied (Fig. 1A, magenta spheres) is distributed in three locations; none of them is located in the active center of the enzyme. The first of the locations, near the C-terminal tail interaction site (Fig. 1A, red), corresponds to the Pro79Leu (P79L), Leu200Pro (L200P) (this report), and Arg219Ile (R219I) variants. Another group of variants, Trp254Leu, Thr256Ile, and Gly257Ser, localize in the core of the protein, in a hydrophobic cluster. Finally, the remaining three variants, Thr228Met, Arg241Cys, and Asp267Gly, are located closer to the enzyme surface.

In the simulation of the wild-type VRK1 protein, the hydrophobic amino acids Leu200 and Pro79 (Fig. 1B, left) closely interact, being located away from the Ile354 residue, which allows some flexibility of the C-terminal tail of the enzyme, facilitating the regulation of auto-inhibition through the cycle of occupancy and release of the active center (ATP site in Fig. 1B). In the case of the Pro79Leu and Leu200Pro variants, the result of molecular dynamics simulation of both variants is a closer contact of Ile354 with Leu79 and Leu200 variant (Fig. 1B, center) or of Ile354 with Pro200 variant (Fig. 1B, right). In both cases, this closer interaction would negatively influence the opening of the active center and thus can affect VRK1 activity.

The VRK1 R387H mutant could not be modeled because it is located very near the end of the C-terminus, and only lacks the final nine amino acids. This R387H variant was also detected in two unrelated Jewish Moroccan patients in homozygosis [36]. This VRK1 C-terminal region has a very low complexity, containing a very flexible basic arginine-rich motif that has several alternative folding conformations, which can regulate the VRK1 kinase activity [34], its targets [37], and protein interactions [33]. This region is also required for the binding of VRK1 to nucleosomes [38]. This nucleosomal interaction with VRK1 is lost in variants located in cluster III, such as R358X lacking this C-terminal region containing the arginine motif [37].

### Protein stability of the L200P and R387H VRK1 variants

Several of the known VRK1 variants have an altered protein stability [39]. Therefore, the protein stability of these two novel variants, L200P and R387H in a compound heterozygous patient, was determined in cells transfected with plasmids expressing each tagged variant, and preincubated with cycloheximide to block de novo translation. The R387H variant was the most stable. The L200P mutant stability was slightly lower than that of the VRK1 wild type (Fig. S1). The kinase-dead VRK1-K179E was used as unstable control (Fig. S1).

### Differential phosphorylation of BAF by L200P, R387H, and other VRK1 pathogenic variants

**Fig. 2:**
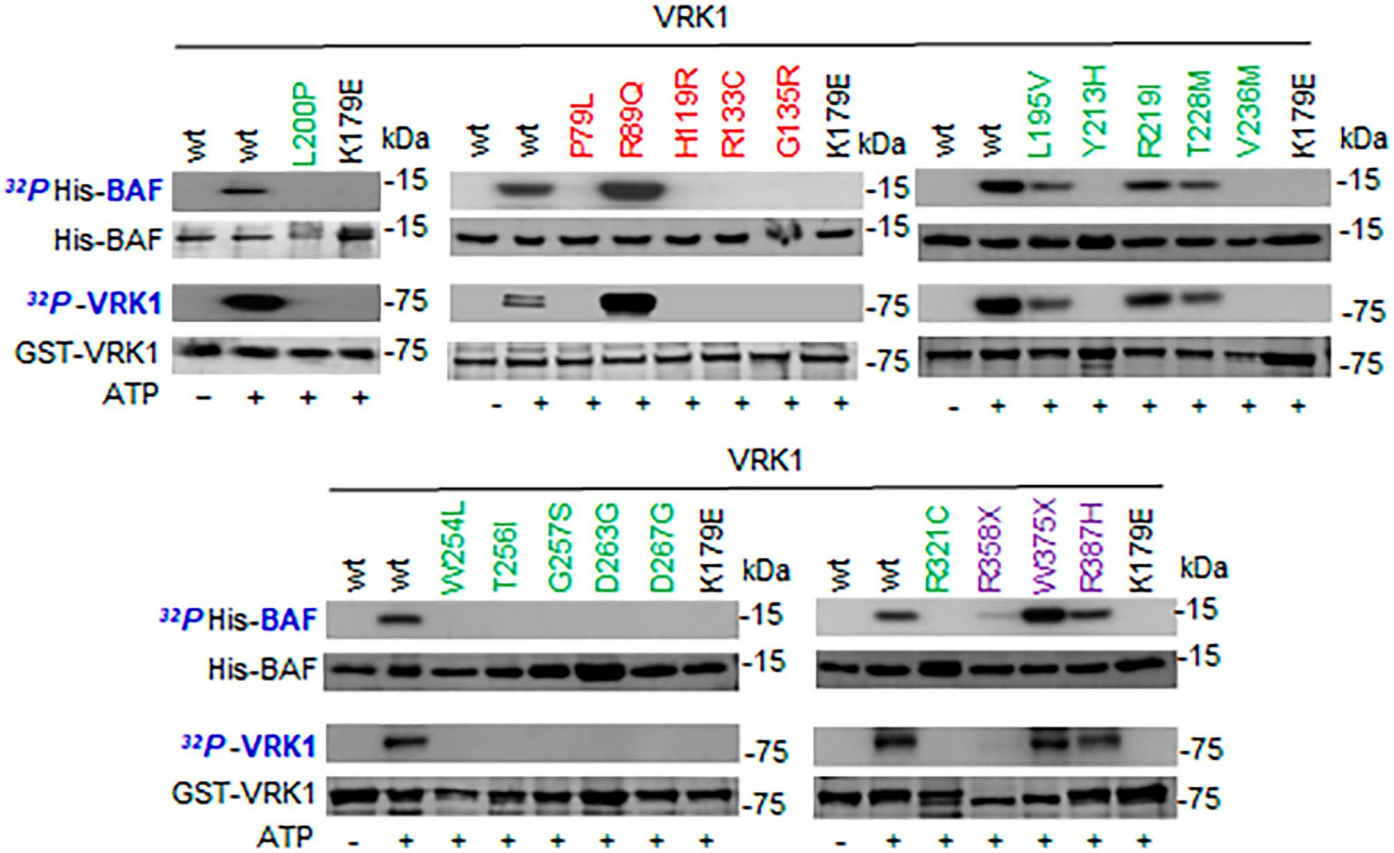
Effect of VRK1 variants on the phosphorylation of BAF. BAF was used as substrate of different VRK1 variants spanning the length of the VRK1 protein and belonging to one of the three clusters of pathogenic VRK1 variants associated with motor neuron diseases. VRK1 autophosphorylation was used as an indicator of its kinase activity. The clusters are indicated by different colors. Red, cluster 1 (next to ATP binding site). Green, cluster 2 (next to kinase catalytic site). Purple, cluster 3 (distal C-terminal low complexity end). The K179E is a kinase-dead protein as negative control

BAF (barrier autointegration factor) is a nuclear protein that facilitates the attachment of chromosomes to the nuclear envelope, and its mutations are associated with progeria [40, 41] and motor neuron diseases [42]. This interaction is regulated by the phosphorylation of BAF by VRK1 [24–26, 43], which causes the detachment of chromosomes from the nuclear envelope and facilitates replication and mitosis, an effect that was initially detected in *Caenorhabditis elegans* [44]. We tested the effect of the two new human L200P and R387H variants, as well as several additional VRK1 pathogenic variants on the phosphorylation of BAF (Fig. 2). L200P and other variants with loss of kinase activity did not phosphorylate BAF, such as P79L, which is close to L200P, H119R, R133C, G135R, Y213H, V236M, W254L, T256I, G257S, D263G, D267G, R321C, and R358X (Fig. 2). However, several VRK1 variants that were kinase active phosphorylated BAF such as R89Q, L195V, R219I, T228M, W375X, and R387H (Fig. 2).

### L200P and R387H VRK1 variants differ on the phosphorylation of nuclear substrates

The L200P and R387H variants are located in two different regions of the VRK1 protein. The L200P variant is located within the mutation cluster 2 after the kinase catalytic site, and the R387H variant is located in the low complexity C-terminal region (cluster 3) that interacts with histones and nucleosomes [38]. These different localizations in the protein sequence might have functional consequences regarding its phosphorylation substrates and interacting proteins [33].

**Fig. 3:**
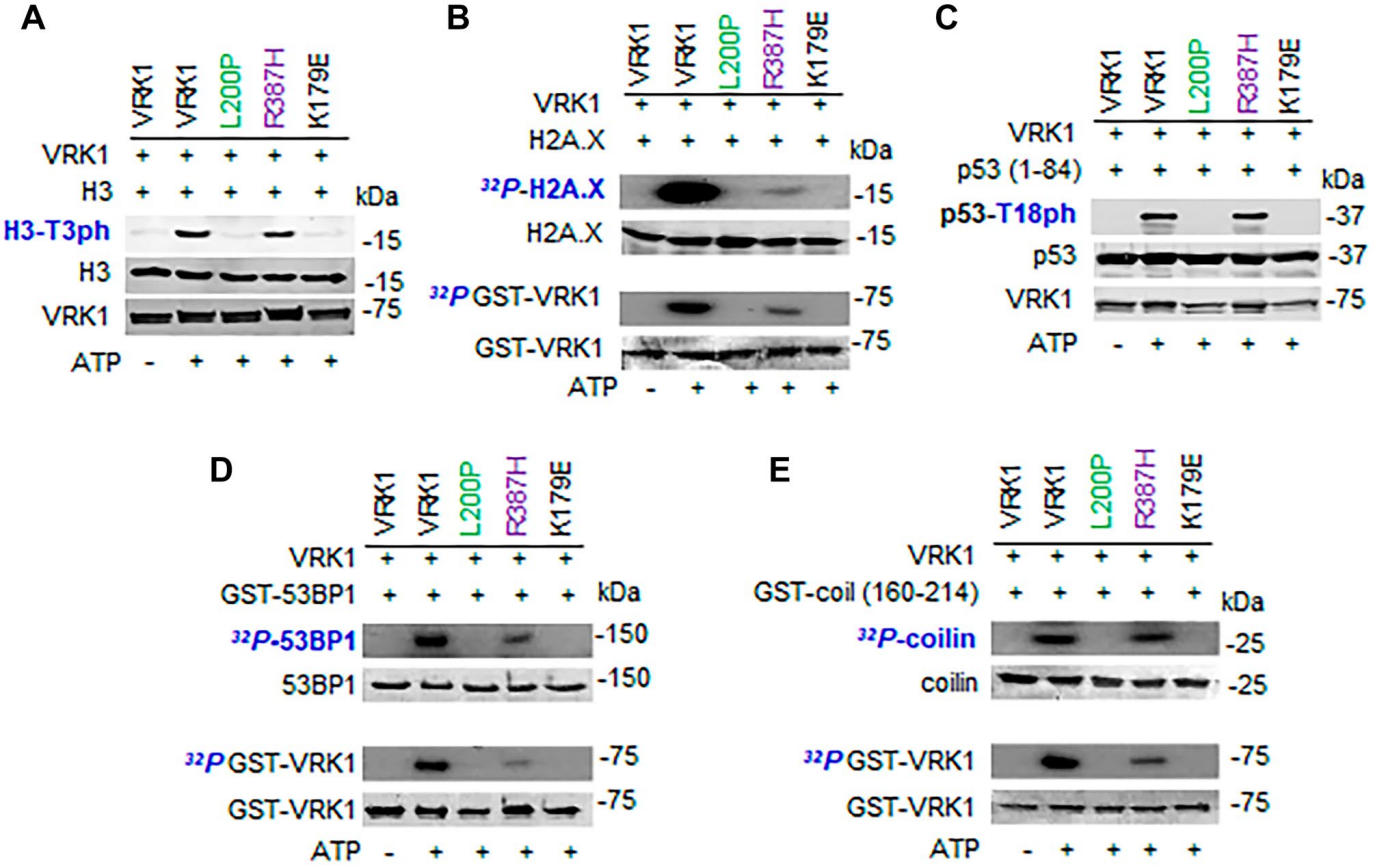
Kinase assays of L200P and R387H VRK1 pathogenic variants on known phosphorylation substrates of VRK1. As controls, wild-type VRK1 and kinase-dead VRK1-K179E were used. **A** Histone H3 phosphorylation. **B** Histone H2AX phosphorylation. **C** P53 phosphorylation is Thr18. **D** 53BP1 phosphorylation. **E** Coilin phosphorylation. In the radioactive kinase assays, the autophosphorylation kinase activity of the variants was also determined. Green, cluster 2 (next to kinase catalytic site). Purple, cluster 3. The K179E is a kinase-dead protein as negative control

Initially, it was determined the effect of the L200P and R358H variants on the phosphorylation of known VRK1 substrates by performing in vitro kinase assays. For these assays, we used as substrates proteins involved in the regulation of different aspects of DNA or RNA biology in nuclei. Therefore, we tested the phosphorylation of histones H3 (Fig. 3A) and H2AX (Fig. 3B), p53 (Fig. 3C), and 53BP1 (Fig. 3D). These proteins are associated with chromatin and DNA damage responses. We also tested the phosphorylation of coilin, a Cajal body protein associated with processes involved in nuclear RNA processing [45]. The L200P variant, located in cluster 2 (next to catalytic site), did not phosphorylate any of these substrates, consequence of the impairment of its kinase activity. In the case of the R387H variant, only H2AX phosphorylation was impaired (Fig. 3B), but the phosphorylation of other substrates, H3, p53, 53BP1, and coilin, was not altered (Fig. 3A–E). This differential effect was also detected in the autophosphorylation of VRK1 that was lost in the L200P variant, but not in the R387H variant. Therefore, these two new VRK1 variants, L200P and R387H, behave differently regarding their kinase activity and substrate specificity (Fig. 3). A similar effect of the variants on the autophosphorylation of VRK1 was detected in radioactive kinase assays (Fig. 3A–E).

**Fig. 4:**
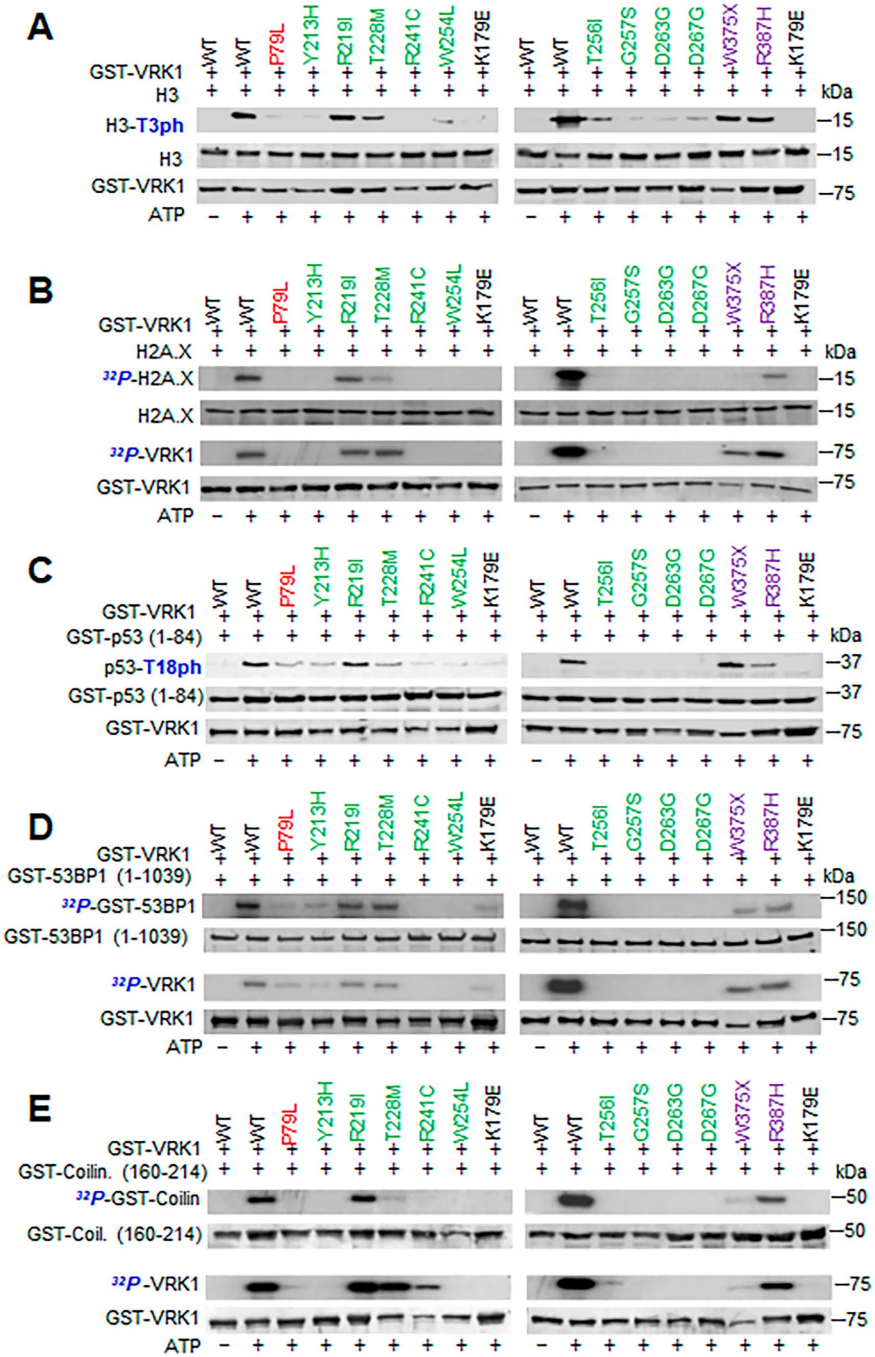
In vitro kinase assays of P97L, Y213H, R219I, T228M, R241C, W254L, T256I, G257S, D263G, D267G, W375X, and R387H. VRK1 pathogenic variants with different known substrates of the wild-type VRK1. As controls, wild-type VRK1 and kinase-dead VRK1-K179E were used. **A** Histone H3 phosphorylation. **B** Histone H2AX phosphorylation. **C** P53 phosphorylation is Thr18. **D** 53BP1 phosphorylation. **E** Coilin phosphorylation. Red, cluster 1. Green, cluster 2. Purple, cluster 3. The K179E is a kinase-dead protein as negative control

Next, we determined the effect of several additional VRK1 protein variants, which were not previously studied, and includes P79L, Y213H, R219I, T228M, R241C, W254L, T256I, G257S, D263G, D267G, W375X (W375*), and R387H, on these substrates in in vitro kinase assays (Fig. 4). The W375* chinese variant, with a termination codon near its C-terminus, was detected in two unrelated patients, one was compound heterozygous with the R387H variant [46], and the other was homozygous [4]. This W375* variant phosphorylates histone H3, p53, and 53BP1, but not histone H2AX nor coilin (Fig. 4). The R219I, T228M, R241C, W254L, T256I, G257S, D263G, D267G, and W375* were modeled as previously described for other VRK1 variants [9, 39].

### VRK1 L200P and R387H variants differently alter H4K16 acetylation in response to DNA damage

**Fig. 5:**
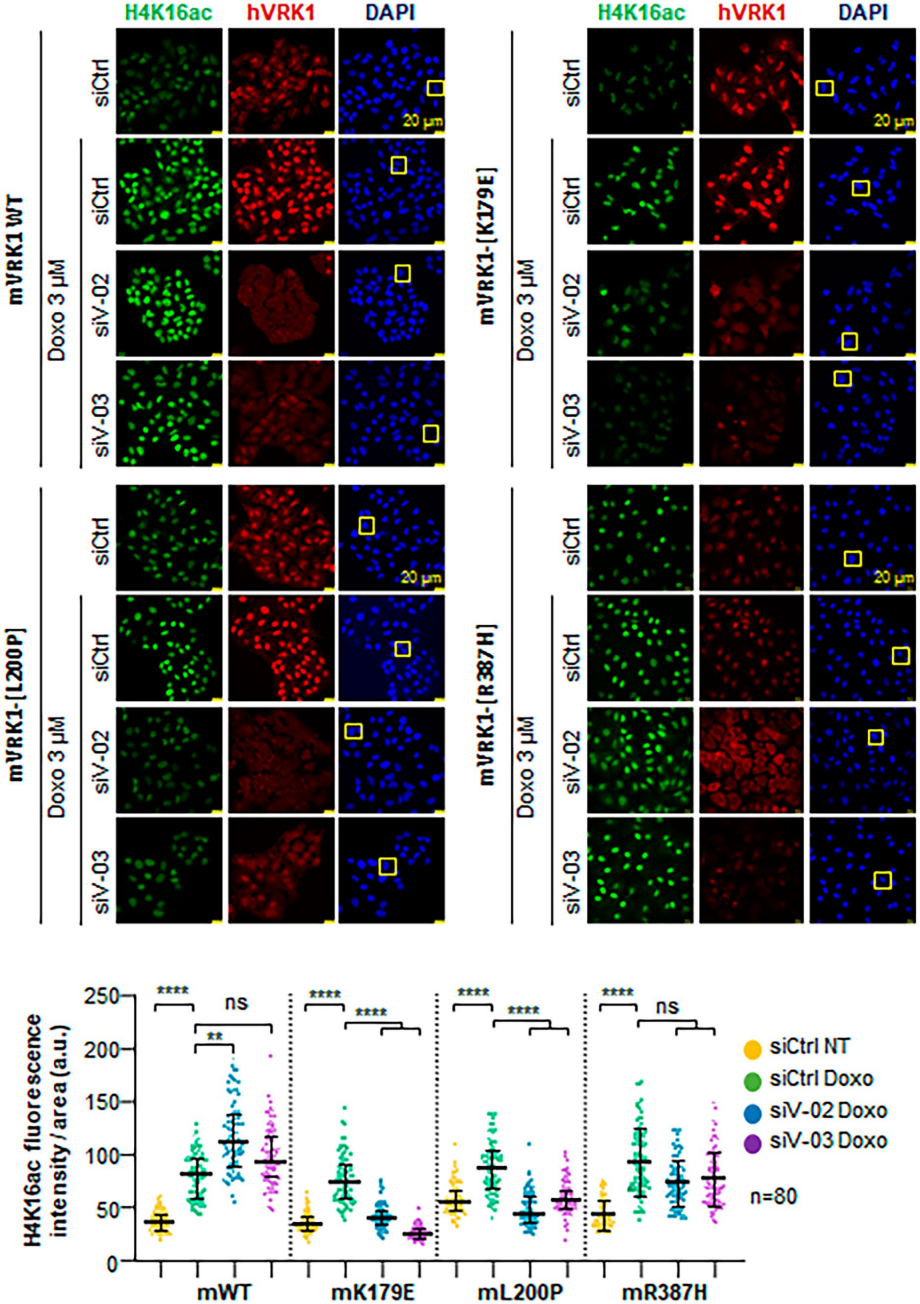
Effect of VRK1 L200P and R387H variants on the acetylation of histone H4 in K16. Immunofluorescence of H4K16 acetylation in stable A549 cell line expressing the indicated murine mutant proteins in which the endogenous human VRK1 was depleted by siRNAs and treated with doxorubicin as indicated. At the bottom, the quantitation of eighty cells is shown. The bar represents 20 µm. WT, wild type; SiCtrl, siControl; siV-02, si-VIK1-02; siV-03, si-VIK1-03; Doxo, doxorubicin

The regulation of histone epigenetic modifications is a major role of VRK1 on chromatin [27, 30]. To study the functional consequences of the two new VRK1 variants, these L200P and R387H variants were introduced in the murine VRK1 (mVRK1) to generate stable cell lines expressing them. In these cell lines, the endogenous human VRK1 was depleted, and the role of the two new variants in the response to DNA damage was determined to test if the variants were able to rescue the loss of functions caused by VRK1 depletion. Doxorubicin treatment induces an accumulation of H4K16 acetylation, which is dependent on VRK1 activity [27, 30]. This H4K16 acetylation was impaired in the cell lines expressing the L200P variant, indicating that this variant was not able to rescue the effect caused by the depletion of the endogenous VRK1 (Fig. 5), and behaves as the kinase-dead K179E used as control, in which histone H4 acetylation was impaired (Fig. 5). However, the catalytically active R387H variant was able to rescue the acetylation of H4K16 in response to DNA damage caused by doxorubicin treatment (Fig. 5).

### Effect of VRK1 pathogenic variants in different regions of the protein on histone H4K16 acetylation

**Fig. 6:**
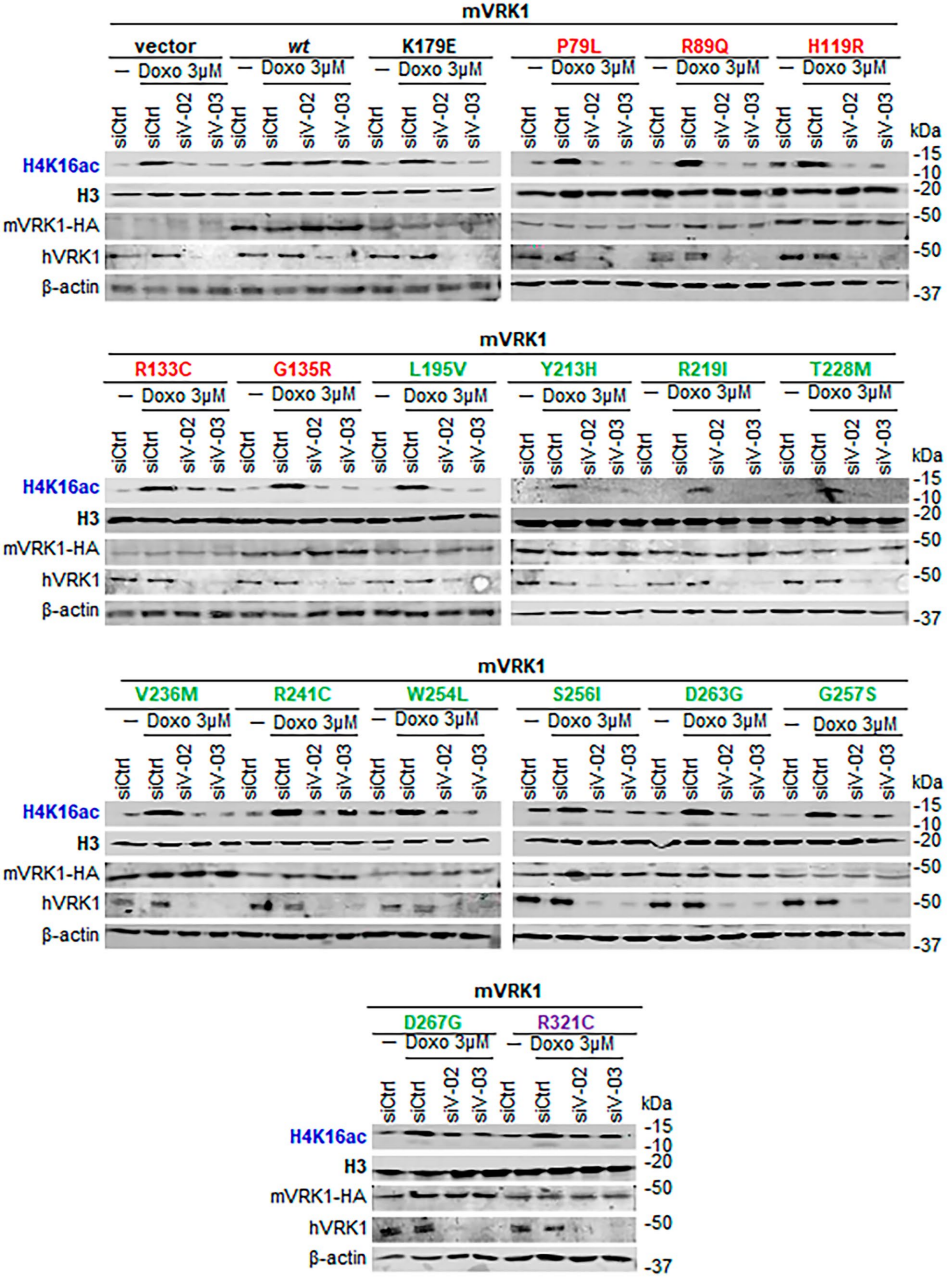
Effect of the VRK1 mutants located in different regions of the VRK1 protein on histone H4K16 acetylation in the response to doxorubicin treatment. Stable A549 cell lines expressing the indicated murine mutant proteins in which the endogenous human VRK1 depleted by siRNAs were used and treated with doxorubicin as indicated. SiCtrl, Si-control; si-V02, si-VRK1-02; si-V03, si-VRK1-02; Doxo, doxorubicin. Red, cluster 1 (next to ATP binding site). Green, cluster 2 (next to kinase catalytic site). Purple, cluster 3 (distal C-terminal low complexity end). The K179E is a kinase-dead protein used as negative control. Immunofluorescence field images are shown in Supplementary Fig. S2, and quantification is in Supplementary Fig. S3

Because the L200P variant impairs histone H4K16ac induced by DNA damage, we tested whether other known VRK1 variants associated with several motor neuron diseases, and not characterized in this context, have a similar effect. This epigenetic modification is critical for the dynamic remodeling of chromatin, and is required for correct DNA damage responses. Several variants are known to impair DNA damage responses detected by their effect on γH2AX [20, 47] and 53BP1 foci formation [7, 9, 39]. VRK1 specifically phosphorylates Tip60 in The158 and Ser199 facilitating both its acetyltransferase activity and translocation to chromatin [27, 30]. Therefore, we tested several VRK1 pathogenic variants belonging to the three different clusters within the VRK1 protein sequence. The following VRK1 variants P79L, R89Q, H119R, R133C, G135R, L195V, Y213H, R219I, T228M, V236M, R241C, W254L, S256I, D263G, G257S, D267G, and R321C were introduced in the murine VRK1 (mVRK1) and tested whether mVRK1 variant proteins were able to rescue the response to doxorubicin in human VRK1-depleted cells. Most VRK1 variants did not rescue H4K16 acetylation induced by doxorubicin, which were detected in immunoblots (Fig. 6). Only two of them, D267G and R321C, were able to rescue the H4K16ac induced in the response to doxorubicin (Fig. 6, bottom panel).

### Effect of VRK1 L200P and R387H on 53BP1 foci in response to doxorubicin

**Fig. 7:**
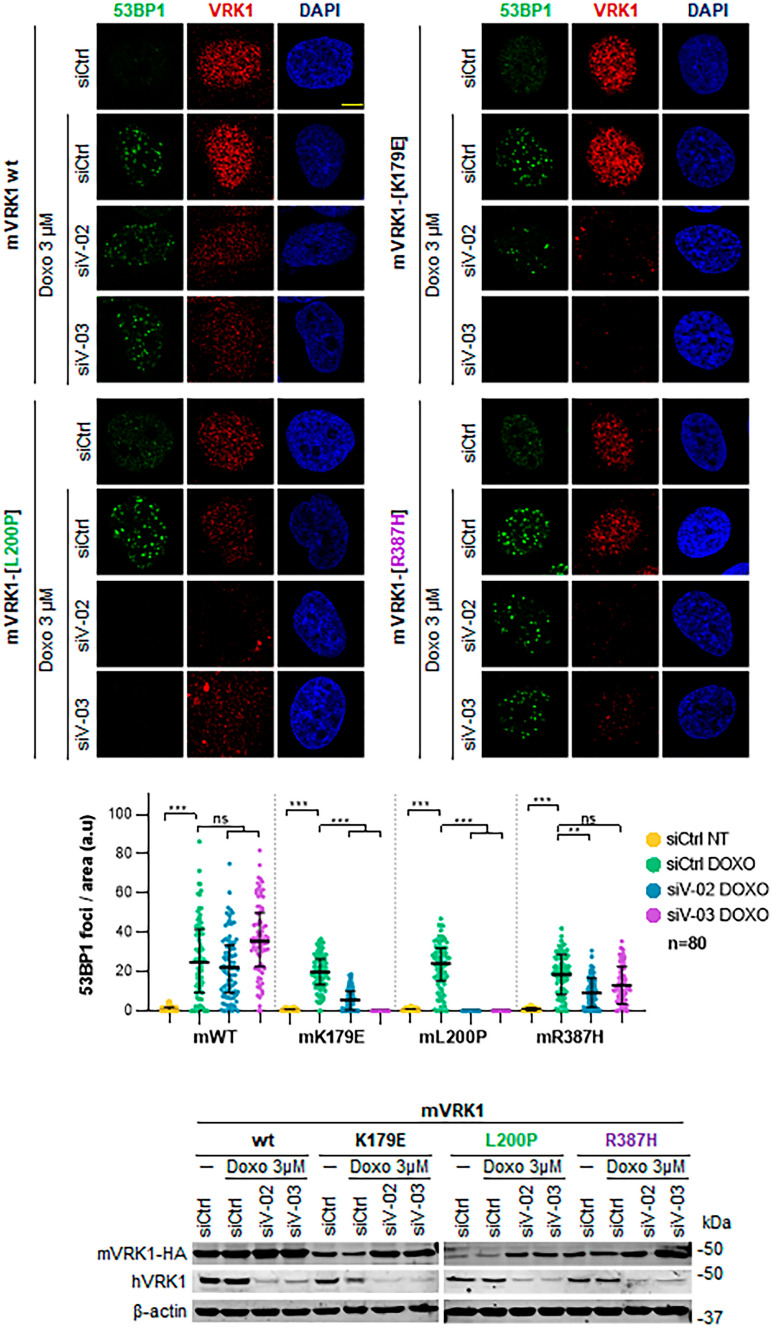
Effect of the L200P and R387H VRK1 variants on the formation of 53BP1 foci in response to doxorubicin treatment. Endogenous human wild-type VRK1 was depleted in cell expression of the murine VRK1 containing the indicated variant. The top panel shows the fluorescence image. Field images are shown in Supplementary Fig. S4. At the bottom, the immunoblots to detected levels of depleted human VRK1 and expressed murine VRK1 variant are shown. ***, *p* < 0.001

A consequence of alterations of H4K16 acetylation will be the impairment of the DNA damage response (DDR), which can be detected by the formation of 53BP1 foci. This has already been shown to be the case for several of the VRK1 variants studied [9, 39, 48]. The formation of 53BP1 foci in response to doxorubicin is regulated by VRK1, by directly phosphorylating 53BP1 in Ser 25/29 [19]. Therefore, the effect of the L200P and R387H variants on the formation of 53BP1 foci induced in response to doxorubicin treatment was determined. The murine VRK1-L200P variant was not able to rescue 53BP1 foci induced by doxorubicin when the endogenous human VRK1 was depleted (Figs. 7 and S4). However, the R387H variant rescued the formation of 53BP1 foci in response to doxorubicin (Figs. 7 and S4).

## Discussion

Bi-allelic VRK1 variants, either homozygous or compound heterozygous, are responsible for a heterogenous group of neurological diseases affecting motor neurons and the peripheral nerves, indicating that the alteration of both alleles is required. This heterogeneity might reflect different, but related functional effects of the variants. In compound heterozygous unrelated patients, all VRK1 variant combinations are unique and different [33, 49].

The P79 and the L200 residues are located very proximal in the VRK1 protein 3D structure, near the ATP binding site, and pathogenic variants in each residue have been individually detected in different patients [50]. The effect of the L200P variant on substrate phosphorylation is similar to that of the P79L variant. These two residues are located very close in the protein structure to the kinase domain, and the variants in either position can impair the kinase VRK1 activity or substrate specificity.

This R387H variant has been detected in patients from three countries, China [46], Israel [36], and Spain [35]. The structural effect of the R387H variant, located in the distal C-terminus of VRK1, is the disruption of the arginine-rich motif and its folding, in a positively charged region that is required for its interaction with both DNA and nucleosomes [38] as well as other nuclear proteins [33]. The R387H variant is able to phosphorylate some proteins such as histone H3, 53BP1, and p53, but H2AX phosphorylation was impaired, which is an early participant in DNA damage responses [51–53].

Patients with variants located in the kinase domain, either near the ATP binding site or catalytic site, do not have neurological pathology in heterozygosis, but have a very severe neurological disease when they are either homozygous, or compound heterozygous. In homozygosis, the neurological diseases start earlier in childhood and in some is prenatal. Both the R387H and W375X (W375*) variants are located in the low complexity C-terminal region, near the end of the protein, and are associated with neurological manifestations of dHMN in middle age patients. In these variants, the VRK1 C-terminal region, required for its interaction with nucleosomes [38], is likely to alter the histone posttranslational epigenetic patterns [14], as well as interactions with other nuclear proteins [33], which are likely to be affected, but have not been studied.

A consequence of VRK1 pathogenic variants in motor neurons is the impairment of their action potential that are smaller in amplitude, larger in duration, and show a more depolarized threshold [54]. These defects in motor neurons, clinically manifested by neurological symptoms, eventually, are the results of the pathogenic roles of the different variant combinations, among them, the effects that VRK1 variants have on the altered assembly and stability of Cajal bodies, which can play a major pathogenic role [3, 7, 9, 23, 37, 39]. Cajal bodies are involved in the correct assembly of ribonucleoprotein complexes, which are exported to cytoplasm, and migrate along neuronal axons to reach synapses [55–57]. If these RNP complexes are defective in an unknown way, it is likely that they may fail during their axonal transport; the longer the axon, the more likely their failure [58]. This is consistent with the initial manifestation of the neurological syndromes in distal parts of the body in patients with pathogenic VRK1 variants. Chromatin alterations can also be a consequence of modifications in the pattern of its epigenetic modifications [32]. The alteration of chromatin relaxation as a consequence of pathogenic VRK1 variants, which impairs histone H4K16 acetylation, can alter not only DNA damage responses, but also the differentiation of neurons during development. These two processes are independent of cell proliferation and can have a pathogenic role in motor neuron diseases. In this context, VRK1 depletion or VRK1 pathogenic variants alter the modification of histone H3 in K9 and K27, by reducing acetylation and increasing methylation. H3K27 methylation is associated with differentiation of spinal motor neurons [59]. The Tudor domain of SMN interacts with methylated histone H3K79 [57]. H3K79 methylation requires the previous acetylation of H4K16 [60], which is also impaired by VRK1 depletion [27, 30].

A subgroup of related and heterogeneous motor neuron diseases is associated with rare homozygous or compound heterozygous VRK1 variants. Patients with the two VRK1 pathogenic variants located in clusters I and II have an early presentation in infancy. Patients with at least one of the variants located in cluster III present a slower disease progression and reach middle age, as is the case in this report and in the W375X (W375*) variant identified in the Chinese population [4, 6]. The R358X (R358*) variant is the only exception; this variant is frequently detected in homozygosis, and generates a truncated protein that is unstable [61], and located in the cytoplasm due to the loss of the nuclear localization signal [39].

The characterization of two new rare VRK1 variants shows that they can also present functional differences regarding their biological effects. Thus, it is likely that different VRK1 protein variant combinations in individual patients will lead to the heterogeneity observed in neurological phenotypes, which are associated with different MND diseases, and other distal neuropathies with neurological manifestations due to different rare VRK1 variant combinations, which individually have differences in substrate phosphorylation and protein interactions.

## Materials and methods

### Clinical case of new *VRK1* heterozygous variants

Two novel variants in the human *VRK1* gene were detected in a juvenile-onset motor neuropathy with pyramidal tract signs in a 49-year-old woman [35]. The patient is compound heterozygous for the two following missense variants in the VRK1 gene (NM_003384.2: c.599 T > C (p.Leu200Pro) (L200P), and c.1160G > A (p.Arg387His/R387H)) [35]. The NM_003384.2:c.1160G > A (p.Arg387His) variant was also reported in dHMN patients from Israel [36] and China [46].

### Structure modeling of human VRK1 missense variants

The three-dimensional (3D) structure of the human VRK1 wild-type protein (Uniprot id: Q99986) was obtained from the Protein Data Bank (PDB id: 2LAV) [34]. Conformer no. 16 (of the 20 NMR conformers included in the PDB file) was selected for further processing. Models for VRK1 variants P79L, L200P were generated using the wild-type structure as template. The initial positions of the side chain of the mutated residues were located using the Pymol Molecular Graphics System (https://pymol.org/; Schrödinger, LLC, Portland, OR) as previously reported [7, 9].

### Molecular dynamics simulation

Structures for wild-type and variant proteins were subjected to 100 ns of unrestrained molecular dynamics (MD) simulation using the AMBER18 molecular dynamics package (http://ambermd.org/; University of California-San Francisco, CA), essentially as previously described [62]. In brief, 3D models were first solvated with a periodic octahedral pre-equilibrated solvent box using the LEaP module of AMBER, with 12 Å as the shortest distance between any atom in the protein subdomain and the periodic box boundaries. Free MD simulation was performed using the PMEMD program of AMBER18 and the ff14SB force field (http://ambermd.org/), applying the SHAKE algorithm, a time step of 2 femtoseconds (fs), and a non-bonded cut-off of 12 Å. Systems were initially relaxed over 10,000 steps of energy minimization, using 1000 steps of steepest descent minimization followed by 9000 steps of conjugate-gradient minimization. Simulations were then started with a 20 picoseconds (ps) heating phase, raising the temperature from 0 to 300 K in 10 temperature change steps, after each of which velocities were reassigned. During minimization and heating, the Cα trace dihedrals were restrained with a force constant of 500 kcal mol^−1^ rad^−2^ and gradually released in an equilibration phase in which the force constant was progressively reduced to 0 over 200 ps. After the equilibration phase, 100 ns of unrestricted MD simulation was obtained for the structures. MD trajectories were analyzed using VMD (Visual Molecular Dynamics) software [63]. Figures were generated using the Pymol Molecular Graphics System (https://pymol.org/. Schrödinger, LLC, Portland, OR).

### Mutagenesis to generate the L200P and R387H VRK1 variants

The amino acid substitutions were introduced in both the human and murine VRK1 cDNA clones using as substrates the following plasmids: pGEX-4 T-VRK1 for bacterial expression and protein purification plasmid [39, 64–66]; plasmid pCEFL-HA-VRK1 [7, 39] and murine plasmid pLenti-C-HA-IRES-BSD-mVRK1 [9]for expression in human cells and rescue experiments. Reaction conditions were previously published [7, 9]. The following primers were used for introducing the amino acid changes: human and murine L200P variant (forward: 5′-TATGGCCCTGCTTATCGGTAC-3′; reverse: 5′-GTACCGATAAGCAGGGCCATA-3′); human R387H variant (forward: 5′-GGAGGCCATACAGACCCACTCAAGAACCAG-3′; reverse: 5′-CTTTCTGGTTCTTGAGTGGGTCTGTATGGC-3′); murine R387H variant (forward: 5′-GGCCGCACAGACCCATTCACGAAC-5′; reverse: 5′-CTGGTTCGTGAATGGGTCTGTGCG-3′); human W375X variant (forward: 5′-GGAACCTGGTGTTGAAGATACGGAATGATCAAACACACAGACAGAGG-3′; reverse: 5′-CCTCTGTCTGTGTGTTTGATCATTCCGTATCTTCAACACCAGGTTCC-3′).

### VRK1 kinase assays

The in vitro kinase assays were performed with [^32^- P]-γATP as previously described [65, 67] or alternatively with the use of specific antibodies to detect the phosphorylation of the substrates as previously reported. For these assays, either GST-VRK1 wild-type and mutants were used [23, 65, 68]. In vitro kinase assays with [^32^- P]-γATP were performed with GST-VRK1 wild-type and variants [23, 65, 68]. Assays with the following substrates were previously published: p53 [69, 70], histone H3 [20, 66], 53BP1 [19], γH2AX [20], and GST-coilin [23] in the kinase reaction buffer for radioactive assays that was composed of (20 mM Tris–HCl pH 7.5, 5 mM MgCl_2_, 0.5 mM DTT, and 150 mM KCl), 5 µM ATP and 5 µCi (0.1 µM) radiolabelled [ɣ-^32^P]ATP, and the indicated substrates in a final volume of 40 µl [7, 9].

### Cell lines, culture, and lentiviral infection

The following validated cell lines were used in this work: A549 (CCL-185) and HEK-293 T (CRL-3216) from the American Type Culture Collection (ATCC, Manassas, VA). Cells were grown in DMEM (Gibco-Life Technologies, Carlsbad, CA) supplemented with 10% fetal bovine serum (FBS), 2 mM de l-glutamine (Gibco-Life Technologies), penicillin (50 u/ml), and streptomycin (50 μg/ml) (Pen/Strep; Gibco-Life Technologies). In the indicated experiments, serum starvation (DMEM supplemented with 0.5% FBS, 2 mM l-glutamine, 1% Pen/Strep) was performed for 48 h.

For the stability assays, HEK-293 T cells were transfected with the corresponding plasmid (pCELF-VRK1-HA wild type or mutated or empty vector) diluted in polyethylenimine (PEI; Polysciences; Warrington, PA, USA) reagent. Forty-eight hours later, 50 ug/ml cycloheximide was added to inhibit protein synthesis. Cells were harvested at the indicated times from cycloheximide addition. Cell extracts were prepared by using a lysis buffer (50 mM Tris–HCl, pH 8.0, 150 mM NaCl, 1% Triton X-100, and 1 mM EDTA) supplemented with protease inhibitors (1 mM PMSF, 10 μg/ml aprotinin, and 10 μg/ml leupeptin) and phosphatase inhibitors (1 mM sodium orthovanadate, 1 mM NaF).

For the study of DNA Damage Response, A549 cell lines expressing murine VRK1 wild type or mutated (kinase-dead K179E, L200P, and R387H) were generated by lentiviral infection. Cells were infected with lentiviral particles containing the vector pLentiC-HA-IRES-BSD (OriGene Technologies, Rockville, MD, USA) expressing murine VRK1 wild type or mutated. Cells were selected with blasticidine (InvivoGen) and cloned by single-cell isolation.

### Immunoprecipitations and immunoblots

Immunoprecipitations were performed using 0.5 to 2 mg of cell lysates in a final volume of 1 ml. Lysates were incubated overnight at 4 °C with the primary antibody according to manufacturer recommendations (1:50–1:500). As negative control, a non-specific antibody was used. Lysates were next incubated with Gammabind plus Sepharose (GE Healthcare) equilibrated with lysis buffer overnight at 4 °C with rotation. The beads were washed by centrifugation at 400 g for 2 min in lysis buffer, and finally the beads were resuspended in loading buffer and fractionated in SDS-PAGE gels.

Proteins were separated in SDS-PAGE gels in running buffer (25 mM Tris–HCl, pH 8.0, 200 mM glycine, 1.7 mM SDS), and transferred to a PVDF membrane (Immobilon-FL, Millipore) in buffer (25 mM Tris–HCl, pH 8.0, 19.2 mM glycine, 15% methanol) as previously described [21, 23, 68, 71]. The primary and secondary antibodies are listed in Tables 1 and 2, respectively. The secondary antibodies were incubated for an hour, and the fluorescence was detected with LI-COR Odyssey Infrared Imaging System.

**Table 1:** Primary antibodies

**Antibody**	**Type**	**Dilution (WB / IF)**	**Supplier**	**Clone / reference**
53BP1	Rabbit polyclonal	— / 1:500	Novus Biologicals	— / NB100-304
Coilin	Rabbit polyclonal	1:1000 / —	Santa Cruz Biotech	— / sc-32860
GST Tag	Mouse monoclonal	1:1000 / —	Santa Cruz Biotech	B-14 / Sc-138
Histone H3	Rabbit polyclonal	1:1000 / —	Cell Signaling	— / 9715
H3-T3ph	Rabbit polyclonal	1:1000 / —	Millipore	— / 07–424
H4K16ac	Rabbit polyclonal	1:1000 / 1:500	Abcam	EPR1004 / Ab109463
HA tag	Rabbit polyclonal	1:1000 / 1:500	Sigma-Aldrich	— / H6908
HA.11 tag	Mouse monoclonal	1:750 / 1:500	BioLegend	901514 / 16B12
p53-Th18ph	Rabbit polyclonal	1:750 / —	Cell Signaling Technologies	— / 2529
VRK1	Mouse monoclonal	— / 1:750	[73]	1B5
VRK1	Mouse monoclonal	1:1000 / —	[73]	1F6
VRK1	Rabbit polyclonal	1:1000 / —	[73]	VC
β-actin	Mouse monoclonal	1:1000 / —	Sigma-Aldrich	AC-15 / A5441
γH2AX	Mouse monoclonal	— / 1:750	Millipore	JBW30105-636

**Table 2:** Secondary antibodies

**Antibody**	**Use**	**Dilution**	**Reference**	**Supplier**
Goat anti-mouse IgG, DyLight 680	WB	1:10000	35518	Thermo Fisher Scientific
Goat anti-rabbit IgG, DyLight 800	WB	1:10000	35571	Thermo Fisher Scientific
Cy2-goat anti-rabbit	IF	1:1000	111–225-244	Jackson ImmunoResearch
Cy3-goat anti-mouse	IF	1:1000	115–165-146	Jackson ImmunoResearch
Cy5-goat anti-mouse	IF	1:1000	115–175-146	Jackson ImmunoResearch
Alexa Fluor Plus 488—goat anti-rabbit	IF	1:1000	A32731	Invitrogen

### Immunofluorescence

Cells were grown in dishes containing coverslips and fixed with 1–3% paraformaldehyde (Sigma-Aldrich) for 30 min at room temperature followed by treatment with 20 mM glycine for 15 min to remove the excess of aldehyde groups. Cells were permeabilized with 0.2% Triton X-100 in PBS 1 × for 30 min, and finally blocked with 1% BSA, and sodium azide in PBS for 1 h at room temperature, or overnight at 4 °C. Next, cells were incubated with the primary antibody at the indicated dilution overnight at 4 °C with 1% BSA and sodium azide in PBS, followed by three washes in PBS. Next coverslips were incubated with the secondary antibody for 1 h at room temperature. Primary and secondary antibodies are listed in Tables 1 and 2, respectively. Cells were incubated with antibodies in darkness to protect the fluorophores. Cells were washed three times in PBS and nuclear DNA was stained with DAPI (4′,6′-diamidino-2-fenilindol) in PBS for 15 min. Coverslips were mounted with MOWIOL-4–88 (Calbiochem). Immunofluorescence was detected with a Leica TCS SP5 or SP8 (Leica Microsystems) microscope and images captured with a Leica DC100 (Leica Microsystems) digital camera. Image analysis was performed using software from LAS AF Lite (Leica Microsystems) and ImageJ (National Institutes of Health).

### Statistical analysis

For statistical analysis, two software packages were used, the IBM SPSS Statistics 23 and GraphPad Prism 8. The following tests were used to compare two groups: ANOVA when the sample has a normal distribution according to the two-tailed Kolmogorov test; and the nonparametric Kruskal–Wallis test when the sample is not a normal distribution. Dates are presented as dot plots. In all cases, the level of significance was 0.05 (*, *p* < 0.05; **, *p* < 0.002; and ***, *p* < 0.001) [72].

### Reagents

Recombinant human histones H3 and H2AX (Millipore, Merck), cycloheximide (Sigma-Aldrich). All other chemicals were from Sigma-Merck (Darmstadt, Germany). Tissue culture media and reagents were from Gibco-Thermo Fisher Scientific (Waltham, MA).

### Supplementary Information

Below is the link to the electronic supplementary material.

Supplementary file1 (PDF 181 KB)

Supplementary file2 (PDF 503 KB)

Supplementary file3 (PDF 719 KB)

Supplementary file4 (PDF 497 KB)

## Data Availability

All data are presented in the manuscript. All material is freely available. No datasets were generated in this report.

## Abbreviations

VRK1: Vaccinia-Related Kinase 1
BAF: Barrier to Autointegration Factor 1
53BP1: TP53-Binding Protein 1.
