# Supplementary material for: Pathogenic effects of Leu200Pro and Arg387His VRK1 protein variants on phosphorylation targets and H4K16 acetylation in distal hereditary motor neuropathy
Source: J Mol Med (Berl). 2024 Mar 30;102(6):801–17. doi: 10.1007/s00109-024-02442-8 (PMC11106162; doi:10.1007/s00109-024-02442-8)
Supplement: Supplementary file 1 — Supplementary file1 (PDF 181 KB) [file 109_2024_2442_MOESM1_ESM.pdf]

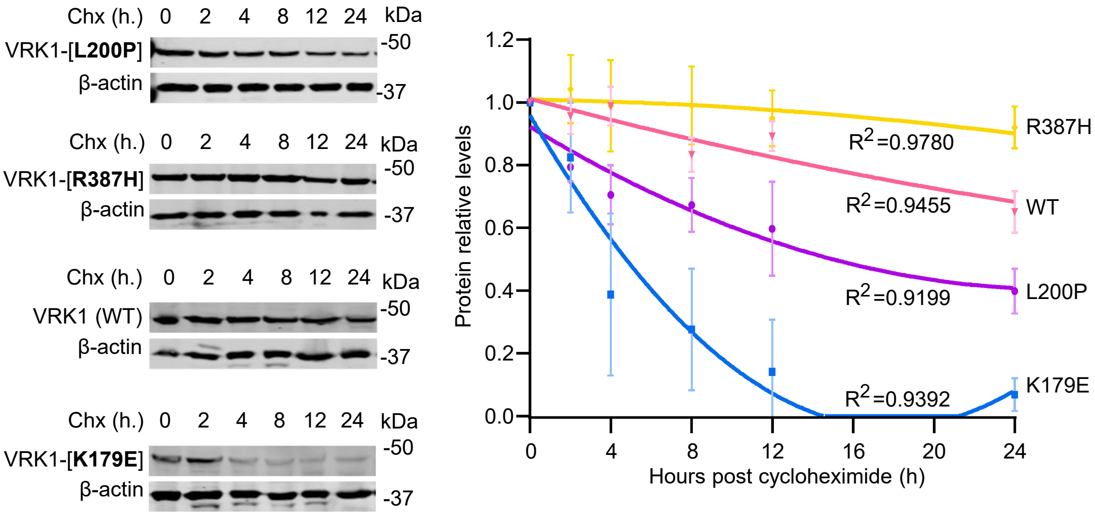

Figure S1. Protein stability of the VRK1 L200P and R387H variants. HEK297T cells were transfected with HA-tagged VRK1 plasmids expressing the variants L200P, and R387H, as well as the wild type and the kinase-dead K179E as controls. The graph shows the quantification of three replicates. WT: wild type.
