# Supplementary material for: Pathogenic effects of Leu200Pro and Arg387His VRK1 protein variants on phosphorylation targets and H4K16 acetylation in distal hereditary motor neuropathy
Source: J Mol Med (Berl). 2024 Mar 30;102(6):801–17. doi: 10.1007/s00109-024-02442-8 (PMC11106162; doi:10.1007/s00109-024-02442-8)
Supplement: Supplementary file 3 — Supplementary file3 (PDF 719 KB) [file 109_2024_2442_MOESM3_ESM.pdf]

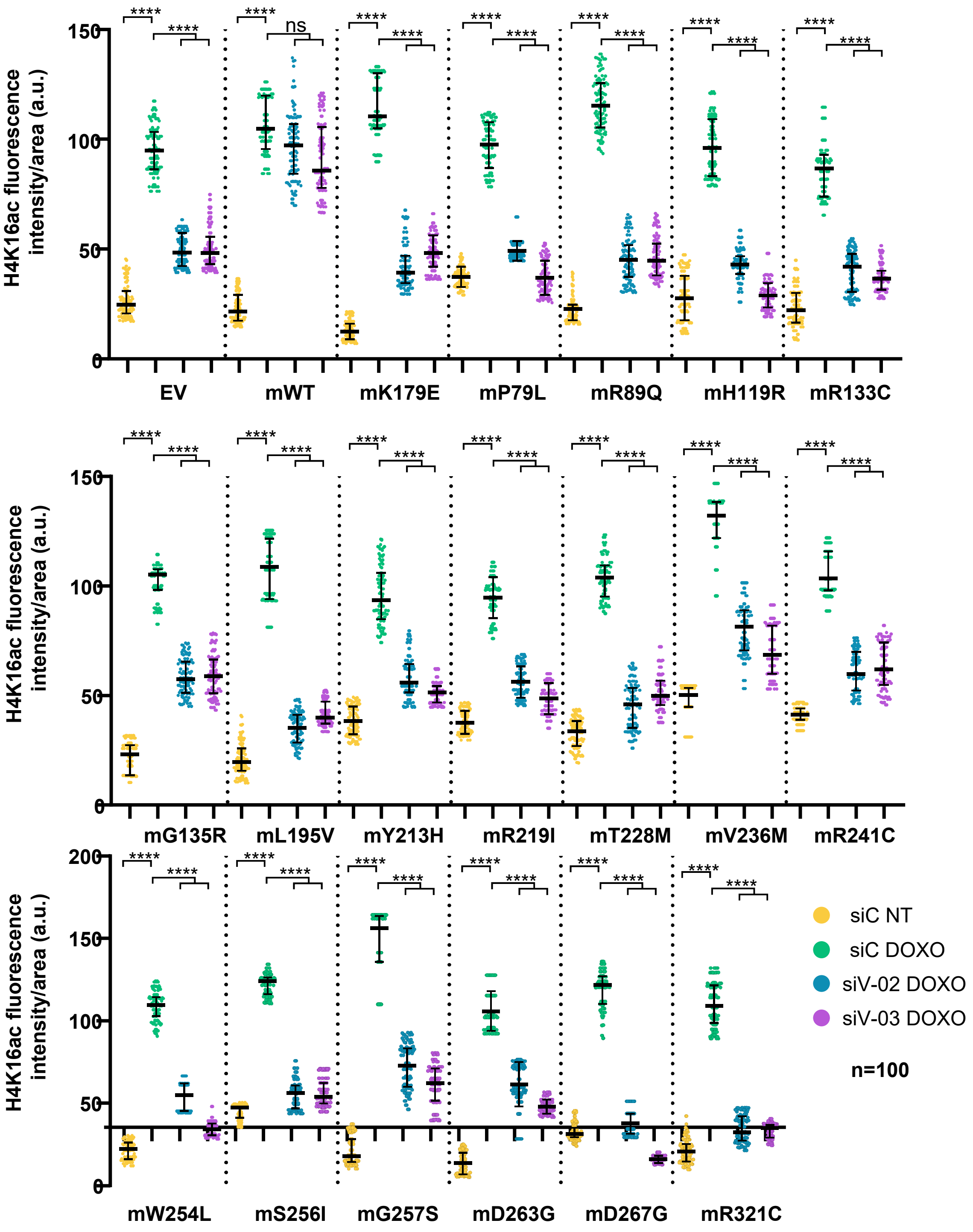

Supplementary Figure S3. Effect of the indicated VRK1 pathogenic variants on histone H4K16 acetylation in response to DNA damage caused by doxorubicin. Stable A549 cell lines expressing the indicated murine mutant proteins in which the endogenous human VRK1 was depleted by siRNAs and treated with doxorubicin as indicated. SiCtrl: Si-control; si-V02: siV-VRK1-02; siV-V03: si-VRK1-02.
