# Supplementary material for: Pathogenic effects of Leu200Pro and Arg387His VRK1 protein variants on phosphorylation targets and H4K16 acetylation in distal hereditary motor neuropathy
Source: J Mol Med (Berl). 2024 Mar 30;102(6):801–17. doi: 10.1007/s00109-024-02442-8 (PMC11106162; doi:10.1007/s00109-024-02442-8)
Supplement: Supplementary file 4 — Supplementary file4 (PDF 497 KB) [file 109_2024_2442_MOESM4_ESM.pdf]

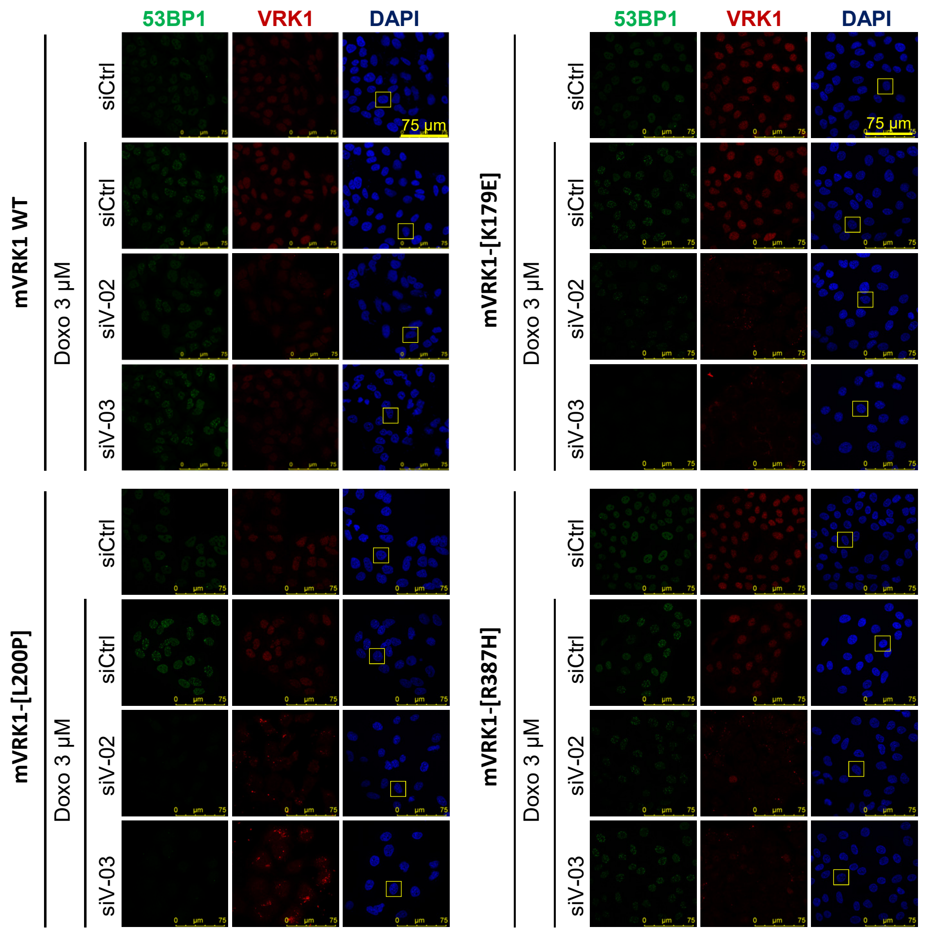

Supplementary Figure S4. Effect of the L200P and R386H VRK1 variants on the formation of 53BP1 foci in response to doxorubicin treatment. Field images of fluorescence. The selected cell shown in Figure 8 is indicated by a box.
